# Sister B., a Memory

**Published:** 1917-10-27

**Authors:** 


### SOME HOSPITAL TYPES OF BRITISH NURSING.

### Sister B., a Memory.

Truly a wonderful woman, who served her hospital faithfully and well for forty years. Her experience extended from the 'sixties, when scrubbers—armed with mysterious bags, in which they carried things both in and out—came to attend to the patients at night, to the present day of modern training-schools. Why our sick forefathers required less attention at night than during the day she could not explain. The "nightbirds" were expected to wash and scrub as well as attend to the needs of patients.

She was an excellent ward sister, always ready to learn new methods and to keep abreast of the times. In her last year of office she attended a special lecture given to probationers by a celebrated surgeon—now a consultant in France—who thanked her for the honour she had done him in coming to listen to his lecture. She had taught him bandaging in the dim past, bandaging which was not only neat and comfortable, but fulfilled its object, which was to keep dressings in their proper place.

No one could beat her at her work. She was an excellent practical teacher. Old resident officers would return after many years and speak gratefully of the help they had always received from her in her large and busy ward. Young men carried their joys and sorrows to her, but woe betide the feckless youth who thought he could teach her probationers some simple detail of nursing, or stood on her freshly hearthstoned step.

It was her custom to give to each member of the honorary medical and surgical staff a small token of goodwill at Christmas, generally a toy. Her last Christmas in hospital she gave an ex-house physician, who had lately started as a consultant, a toy motor-car; a wag exchanged the motor for a neat donkey and cart. The recipient called on Sister and thanked her, a little coolly, for her gift. She, suspecting nothing, said "I am glad you liked it, sir, and hope you will drive one yourself before long."

When a new matron came and made alterations, as new matrons will, she brought her sisters on duty half-an-hour earlier in the morning, and offered to make an exception in the case of Sister B. on account of long service; but she was told, when sisters, old or young, could not carry out orders it was time for them to retire. In every way this old sister supported her new chief and made her feel she had a loyal helper who would, as far as it lay in her power, do what she could to make her rather difficult path as smooth as possible. We shall never look upon her like again.

Many will recognise this well-known sister, and will be glad to know she retired on a comfortable pension, and lived in a delightful cottage near her old hospital. She often visited her old home but rarely her old ward, and only lived six years after her retirement. She was well over seventy when she died. "Ward One."

